# On the Robustness of Quantum Algorithms for Blockchain Consensus

**DOI:** 10.3390/s22072716

**Published:** 2022-04-01

**Authors:** Muhammad Asad Ullah, Jason William Setiawan, Junaid ur Rehman, Hyundong Shin

**Affiliations:** Department of Electronics and Information Convergence Engineering, Kyung Hee University, Yongin-si 17104, Gyeonggi-do, Korea; asad@khu.ac.kr (M.A.U.); jasonwilliam98@khu.ac.kr (J.W.S.); junaid@khu.ac.kr (J.u.R.)

**Keywords:** blockchain consensus, quantum algorithms, quantum noise, distributed sensor networks, modular computing units

## Abstract

Blockchain has revolutionized many fields, such as distributed sensor networks, finance, and cryptocurrency. Consensus between distributed network nodes is at the core of such blockchain technologies. The three primary performance measures for any consensus algorithm are scalability, security, and decentralization. This paper evaluates the usefulness and practicality of quantum consensus algorithms for blockchain-enhanced sensor, and computing networks and evaluates them against the aforementioned performance measures. In particular, we investigate their noise robustness against quantum decoherence in quantum processors and over fiber-optic channels. We observe that the quantum noise generally increases the error rate in the list distribution. However, the effect is variable on different quantum consensus schemes. For example, the entanglement-free scheme is more affected than entanglement-based schemes for the local noise cases, while in the case of noisy optical fiber links, the effect is prominent on all quantum consensus schemes. We infer that the current quantum protocols with noisy intermediate-scale quantum devices and noisy quantum communication can only be employed for modular units in intraenterprise-level blockchain, such as Zilliqa, for sensor, and computing networks.

## 1. Introduction

Blockchain refers to a system of distributed ledgers recording information that maintains the fidelity, security, and trust of data without needing a trusted third party. Blockchain provides security, privacy, and trust management, essential for distributed technologies such as sensor networks [1]. At the heart of these provisions is the consensus in blockchain networks.

Consensus algorithms constitute an agreement between distributed network nodes on a value or decision. They are at the core of cooperative networks and determine their performance to an extent [2]. Depending on a distributed network’s specific nature and resources, its consensus algorithm can be tolerant against crash and/or malicious attack failure. The consensus algorithms that can tolerate malicious attacks are broadly classified under the umbrella of the Byzantine Agreement (BA) [3]. BA refers to the problem of nonfaulty parties in the network agreeing on the same decision in the presence of up to 1/3rd malicious parties [3]. The BA problem is solvable only if each participant in the consensus holds a private list suitably correlated to the private lists held by its peers but unknown to them. Therefore, the problem of BA consensus can be reduced to the problem of sharing private lists in the network [4].

Quantum resources provide many nonclassical features that have been utilized for groundbreaking works in secure communication. Researchers have recently provided many quantum algorithms to achieve BA using quantum resources such as entanglement and coherence. The first quantum protocol is the elegance of Aharonov states for generating six-combination private lists in a tripartite network [5]. Iblisdir et al. the used Quantum key distribution (QKD) to achieve the same lists through two quantum channels in the same classical network configuration [6]. Later protocols on higher dimensional entanglement focused on simplifying the BA procedure by providing four-combinations private lists [4,7,8,9]. A few quantum protocols have been proposed in recent years that achieve BA without entanglement [10,11,12]. If the BA condition is relaxed to include the option of all loyal parties aborting, the quantum BA schemes can tolerate up to half faulty participants in the network. Hence, unlike classical methods, they can provide an agreement for a tri-partite network with one faulty node. These consensus mechanisms have been employed for quantum tasks such as clock synchronization and secret sharing [11,13,14].

Currently, most blockchains employ classical BA-based practical Byzantine fault tolerance (PBFT) algorithm or its derivatives for countering malicious nodes in blockchain consensus networks [15]. QBA schemes allow better fault tolerance than PBFT and allow lower communication complexity, making them promising candidates for blockchain consensus. However, in the noisy intermediate-scale quantum (NISQ) era, their performance has not been evaluated for this task in a practical network setting.

In this paper, we evaluate the performance of various quantum Byzantine agreement (QBA) protocols in terms of their scalability, security, and decentralization for blockchain consensus. In particular, we investigate the robustness of these algorithms for practical NISQ devices. The main contributions of this paper can be summarized as follows.

- *Realization of Practical NISQ Era QBA Networks:* Quantum schemes provide better fault tolerance and communication complexity; however, they will be prone to quantum noise in the NISQ era devices. For consideration of these quantum algorithms for practical blockchain networks, quantum noise in the networked setting is inevitable. Furthermore, no theoretical analysis of the quantum noise exists for these QBA schemes to date. Therefore, there is a need to investigate these QBA algorithms in practical NISQ era networked settings for the aforementioned performance measures. In particular, the analysis of such algorithms for noisy quantum processor memory and noisy quantum channels for different noise models and noise levels is essential so we can utilize them for practical blockchain consensus. For this purpose, we consider qubit decoherence and depolarizing noise in quantum processors and noisy quantum fiber-optic channels and evaluate the performance of QBA algorithms.
- *Emulation of QBA Networks for Near-Practical Applications:* Quantum computers and simulators have existed for some time now, but there were no quantum networks available for implementing quantum network algorithms until recently. Although we can simulate network protocols like teleportation on quantum computers/simulators, they do not carry the essence of network settings such as impacts of the quantum handshake, fiber-optic delays, and change of qubit modality from static to flying qubits. Chien et al. used a localized experimental setup to demonstrate the QBA; however, it does not provide information about its practicality in a networked setting for Blockchain consensus [16]. Moreover, the experimental settings in [4,13] provide results for a particular setting of node distance, noise model, and the number of parties and cannot be used for generalized results. Recently, researchers at QuTech proposed a discrete event quantum network simulator (NetSquid) which allows emulating network applications while considering the impacts of practical considerations on them [17]. Netsquid is currently being augmented to an actual quantum network through the *Quantum Network Explorer* [18]. We have used Netsquid to emulate the practical NISQ era QBA systems as close as possible to practical experiments.
- *Consideration of QBA in the Absence of Quantum Handshake:* Quantum devices require shared phase definitions in addition to sharing the reference frames [19]. This need is a critical bottleneck for distributed quantum computation, clock synchronization, and QKD networks [19,20]. In practical settings, the phase definitions of quantum devices can change over time, leading to a nonfungible source of quantum decoherence. We not only show how this affects QBA systems but also identify which QBA algorithms are best suited for this problem.
- *Performance Evaluation of QBA Algorithms:* We evaluate the error rates in list distribution for each QBA protocol and compare their performance. We observe that quantum consensus algorithms have better malicious fault tolerance (security), lower scalability, and comparable decentralization when compared to their classical counterparts.

## 2. Quantum Consensus Algorithms

To compare different QBA algorithms, we first discuss the entanglement-based schemes for list distribution before discussing entanglement-free scenarios that utilize quantum coherence. We will evaluate the performance of these protocols in terms of the security, scalability, and decentralization for blockchain consensus.

### 2.1. Entanglement-Based Quantum Consensus

The first quantum BA algorithm utilized three-qutrit Aharnov state shared between the three participants [5]

(1) $$\begin{array}{cc} |\psi \rangle & =\frac{1}{\sqrt{6}}\left(|0,1,2\rangle +|1,2,0\rangle +|2,0,1\rangle \right.\;\\ \; & -|0,2,1\rangle -|1,0,2\rangle -|2,1,0\rangle ). \end{array}$$

However, it utilized qutrits which are not readily available and controllable in the NISQ era devices. Therefore, for NISQ era quantum networks, we will consider only qubit-based procedures [21].

#### 2.1.1. QKD-Based QBA

QKD is the protcol for distributing secret keys between two parties [22]. Two QKD channels between three parties can share private correlated lists between them. For this purpose, we consider the E91 protocol based on shared enlargement due to its superior performance over the original BB84 scheme [22,23]. In this case, Alice prepares two copies of an entangled state

(2) $$\begin{array}{c} |\psi \rangle =\frac{1}{\sqrt{2}}\left(|00\rangle +|11\rangle \right). \end{array}$$

She keeps one particle of each copy to himself and shares the other particle of the first (second) entangled state to Bob${}_{1}$ (Bob${}_{2}$) over the optical fiber. Once entanglement distribution has been established, each party applies local measurement on their local qubits in either the computational (Pauli-Z) or the diagonal (Pauli-X) basis. Similar to QKD schemes, the parties then reveal their measurement basis, and if the bases agree, the private lists correspond to the measurement results.

#### 2.1.2. Singlet-Based QBA

For singlet-based QBA, a quantum source distributes four-qubit singlet states [4]

(3) $$\begin{array}{cc} |\phi \rangle & =\frac{1}{2\sqrt{3}}\left(2|0011\rangle -|0101\rangle -|0110\rangle \right.\;\\ \; & -|1001\rangle -|1010\rangle +2|1100\rangle ). \end{array}$$

to the participants of the QBA. Alice receives two qubits while Bob${}_{1}$ and Bob${}_{2}$ receive one qubit each. Each party chooses a measurement basis and applies it to its local qubits. After measurement, all parties announce their measurement bases. If their measurement bases are the same, the measurement outcomes become private list entries. This singlet-based bit-valued decision was recently upgraded to multivalued BA through a *d*-dimensional entangled system [24].

From the perspective of decentralization, both entanglement-based systems involve local measurement by each of the participants; hence, they are fairly decentralized. Regarding security, the entanglement distribution procedure halts if an adversary intercepts a particle in the entangled states in (2) and (3) over the channel. Furthermore, the QKD-based scheme utilizes the unconditional security of the quantum cryptographic schemes. Therefore, these schemes outperform their classical counterparts in terms of security. In terms of scalability, the entangled states are highly susceptible to channel noise, and the loss of a single particle from singlet or GHZ states leads to the entire distribution being rendered useless. As the number of participants increases, these schemes are increasingly prone to failure.

### 2.2. Entanglement-Free Quantum Consensus

We now investigate the private correlated list distribution protocol using an unentangled qutrit or qudit to achieve BA [11,13,25]. This protocol utilizes a single *K*-dimensional qudit

(4) $$\begin{array}{c} |\xi \rangle =\frac{1}{\sqrt{K}}\sum_{k=0}^{K-1}|k\rangle , \end{array}$$

where the number of dimensions corresponds to the number of parties involved in the scheme. The first node (Alice) prepares the qudit, encodes her choice of basis b through unitary encoding

(5) $$\begin{array}{c} V=|0\rangle \langle 0|+\sum_{k=1}^{K-1}e^{\dot{\iota }2\pi b/K}|k\rangle \langle k|, \end{array}$$

and her choice of secret private list entry s through

(6) $$\begin{array}{c} U=\sum_{k=0}^{K-1}e^{\dot{\iota }2\pi ks/K}|k\rangle \langle k|, \end{array}$$

respectively, and sends it to the second party (Bob${}_{1}$). The second party similarly encodes its choices and sends the qudit to the next Bob${}_{2}$. Once the last party, Bob${}_{K-1}$, receives the state, he applies its encoding and measures the qudit in Fourier basis {|ξ〉〈ξ|,I−|ξ〉〈ξ|. If the measured result is |ξ〉, each party reveals the basis choice $b_{i}$∀i∈K in random order. If the choice of basis $b_{i}\;\mathrm{mod}\;K=0$, the private list entries are correlated.

The advantage of using a qudit-based scheme is that it increases the scalability of the network compared to entanglement-based methods because it employs only one detector. However, this also leads to lesser decentralization because only a single party performs the measurement. Furthermore, the single qudit traveling over the channel is more susceptible to malicious attacks due to the entire unentangled quantum system available to the adversary, thereby reducing its security [26].

#### Multi-Qubit Implementation of Qudit-Based QBA

As discussed in the previous section, qudit-based QBA is the most practical QBA candidate due to its better scalability. However, for the current era of noisy intermediate-scale quantum (NISQ) devices, generation and manipulation of qudits suffer from practical constraints, including unavailability and limited control [17,27]. Therefore, we utilize a multiqubit system that mimics the qudit with similar network architecture. To generate the state (4) for *K*-partite system, we use $\left\lceil \log_{2}K\right\rceil$ qubits, where the each qubit is in the maximal superposition state $\frac{1}{\sqrt{2}}\left(|0\rangle +|1\rangle \right).$ The U operator is decomposable to single-qubit operations but the V operator requires joint evolution of all qubits at the local processor. For implementation on the NISQ era quantum network, we consider that the qubits both arrive at the same time and that the processors can employ two-qubit operations.

## 3. Quantum Noise in NISQ Networks

For the NISQ era, quantum noise has been the fundamental bottleneck in implementing practical quantum computation and communication systems. For practical realizations of consensus algorithms, quantum noise will degrade the quality of the qubits over the channel and in the quantum processor memory. The noise can degrade speakable and/or unspeakable information carried by the quantum processor network. In this section, we investigate the performance of the three qubit-based QBA protocols against both unspeakable (nonfungible) and speakable (fungible) quantum noise [19]. The nonfungible quantum noise is the absence of shared quantum phase reference between the distributed processors, an additional reference frame required for distributed quantum computation alongside the shared inertial reference frame, while speakable quantum noise constitutes channel and local quantum noise affecting the qubit’s information-carrying degree of freedom over the optical fiber or local quantum processor memory. Our analysis is limited not only to quantum networks but also encompasses the investigation of the QBA schemes for blockchain networks.

### 3.1. Quantum Noise in Nonfungible Information—Absence of Shared Phase Reference

**Figure 1:**
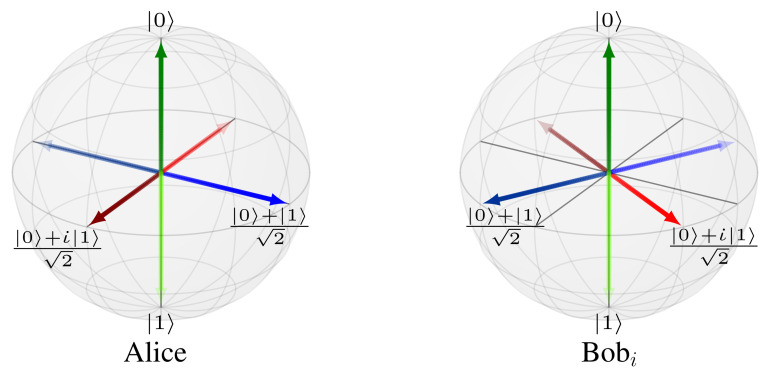
Absence of shared phase reference between Alice and Bob${}_{i}$.

Shared phase reference refers to the existence of common definitions of superposition quantum states, e.g., $|+\rangle =\frac{1}{\sqrt{2}}\left(|0\rangle +|1\rangle \right)$, and therefore the definitions of local operators such as Hadamard operation

$$H=\frac{1}{\sqrt{2}}\left(\begin{array}{cc} 1 & 1\\ 1 & -1 \end{array}\right),$$

for quantum information processing at distributed nodes (see Figure 1). The absence of shared phase reference becomes a problem if the quantum system is prepared at one party and undergoes controlled evolution under nondiagonal operators or noncomputational basis measurement at the other parties.

For the entanglement-assisted quantum consensus schemes, one party (say Alice) prepares the entangled state locally and then shares one particle of each entangled state with Bob [20,25]. Each party measures the qubit, either in the Pauli-Z or Pauli-X basis at random. Recently, entanglement purification has been identified as a possible solution to counter the absence of shared phase reference for entanglement distribution [20,28]. In this procedure, using the quantum circuit method, Bennet et al.’s entanglement purification is iteratively used to obtain singlets in the local basis [28]. However, it requires *n* entangled states and leads to the loss of half population of entangled states in each purification cycle. Furthermore, the fidelity $F_{n}$ of the entangled pairs after *n* rounds of purification is [28]

(7) $$\begin{array}{c} F_{n}=\frac{F_{n-1}^{2}+\frac{1}{9}{\left(1-F_{n-1}\right)}^{2}}{F_{n-1}^{2}+\frac{2}{3}F_{n-1}\left(1-F_{n-1}\right)+\frac{5}{9}{\left(1-F_{n-1}\right)}^{2}}, \end{array}$$

where $F_{n-1}$ is the fidelity after the $\left(n-1\right)$th round of purification. After the *n*th round, the *X* entangled states are *reduced* to $X/2^{n}$ singlets. Furthermore, the probability of discarding the purification results for a round is *nonzero.*

For the entanglement-free scheme, the qubits are prepared locally at Alice and undergo diagonal operator evolution at other parties. The last party measures the multi-qubit state in the Fourier basis, which is a non-computational-basis measurement. Therefore, the absence of shared phase reference (SRF) becomes a problem.

### 3.2. Quantum Noise in Fungible Information

In addition to the desynchronization of phase reference, the quantum processor and fibre-optic links may have quantum noise causing qubit decoherence. We consider two of the most common models of local processor and fibre-optic quantum noise:

- Qubit decoherence (Finite dephasing time):
  For NISQ quantum networks, a qubit residing in quantum memory or in transit over fiber-optic channel undergoes dephasing for the duration of its stay/transit. For a qubit in the state ρ, it undergoes the noisy transition to
  (8) $$\begin{array}{c} \sigma =(1-p)\rho +pZ\rho Z, \end{array}$$
  where *Z* is the Pauli-Z operator on the qubit and *p* is the dephasing noise parameter.
- Depolarizing noise:
  Depolarizing noise is the worst form of quantum noise that includes decoherence along all three Pauli axis in the Bloch sphere. For a qubit in the state ρ, it undergoes the transition to
  (9) $$\begin{array}{c} \xi =(1-p)\rho +p\pi , \end{array}$$
  where π=I/2 is the maximally mixed qubit state and *p* is the depolarizing noise parameter.

## 4. NISQ Network Setup for QBA Algorithms

We now investigate the noise-robustness of the three qubit-based QBA schemes. For our analysis, we utilize the NetSquid network simulator for evaluating the error rate in list distribution for QBA [17]. Netsquid is explicitly built to emulate a quantum network. It models the network by NISQ-era quantum devices connected by fiber-optic links for classical and quantum information transfer. Utilizing the Netsquid, we can capture the protocol’s behavior as close to the actual practical implementation as possible instead of simplistic localized simulation using a quantum or classical computer. Recently, Chien et al. utilized a general quantum circuit simulator to simulate the Byzantine agreement. This result, however, is obtained by running the circuit locally using static qubits such as superconducting qubits, ignoring any communication-related issues. Furthermore, with the development of *Quantum Network Explorer* by the same team, soon we will be able to implement NetSquid emulation in an actual open-source quantum network directly [18].

**Figure 2:**
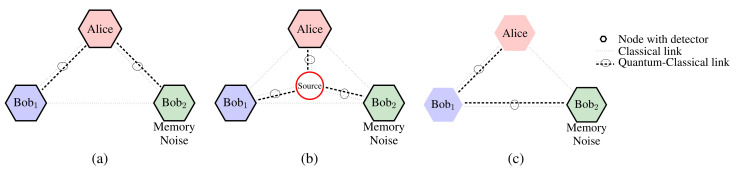
Network architecture for three QBAs: (**a**) QKD-based QBA, (**b**) singlet-based QBA, and (**c**) multiqubit-based QBA. The classical links are pairwise, while quantum links form a chain. We assume that Bob${}_{2}$ can have local memory noise.

We have analyzed our results for tripartite networks with the aforementioned noise models associated with the quantum memory and channels. The tripartite case is the most common consideration for the Byzantine agreement problems, in which the quantum algorithm can solve QBA if one party is faulty, unlike classical algorithms. By solving the three-party case, the solution can be generalized to an arbitrary number of parties with t<n/2 fault tolerance, where *t* denotes the number of faulty parties and *n* denotes the total number of parties. Figure 2 shows the network connection of QKD, singlet-based, and multiqubit-based schemes that we deploy in the NetSquid network simulator and provide the codes in the *Github repository* in [29]. The simulation parameters are listed in Table 1.

We model quantum and classical connections as fiber-optic channels that experience propagation delay, depolarizing, or dephasing (the most practical decoherence models). The length of both classical and quantum are assumed to be four meters as we are considering a modular computing setup where each quantum processor is separated with a relatively small distance; hence, we do not consider any use of quantum repeater in the simulation. For noise in processor nodes, we consider that Bob${}_{2}$ lacks shared phase reference with other nodes alongside the localized qubit depolarizing or dephasing. We run the protocol until we obtain a list with a length of 100 entries for all parties for each noise parameter value *p* ranging from 0 to 1 and plot the average error rate against that particular noise value for 100 data average.

## 5. Discussion

**Figure 3:**
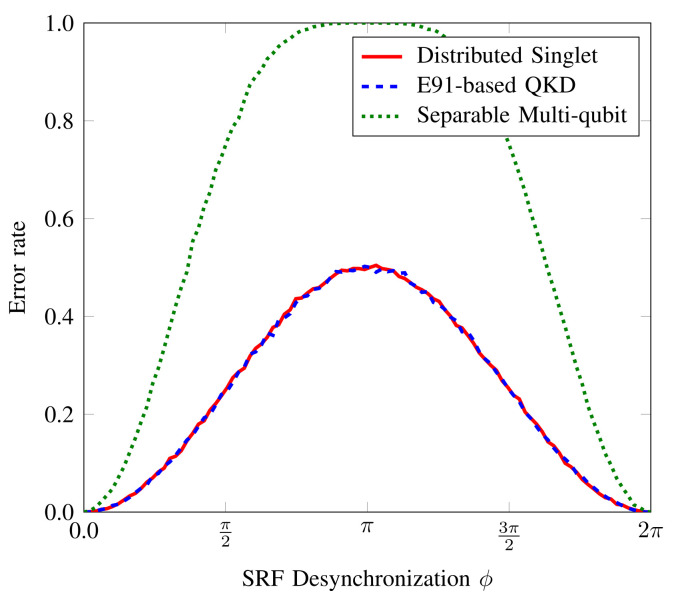
Impact of the absence of shared phase reference on the list distribution in tri-partite network.

Figure 3 shows the error rate in the private correlated lists as the phase definitions of Bob${}_{2}$ desynchronize with other parties. As the desynchronization in the phase definitions increases, the error rate increases. The increase in error rate of multiqubit scheme is more adverse because all qubits undergo the effects of desynchronized phase definitions when measured, unlike entanglement-based QBA schemes where qubit measured at Bob${}_{2}$ is the only one experiencing noise effects. It becomes maximum for the case where the *X*-axis of Bob${}_{2}$’s Bloch sphere aligns with the *Y*-axis of Alice and Bob${}_{1}$’s Bloch sphere. From thereon, due to the circular nature of phase definition, the error begins to reduce, and for phase desynchronization 2xπ,∀x∈Z, the phase definitions are synchronized and lead to no error.

**Figure 4:**
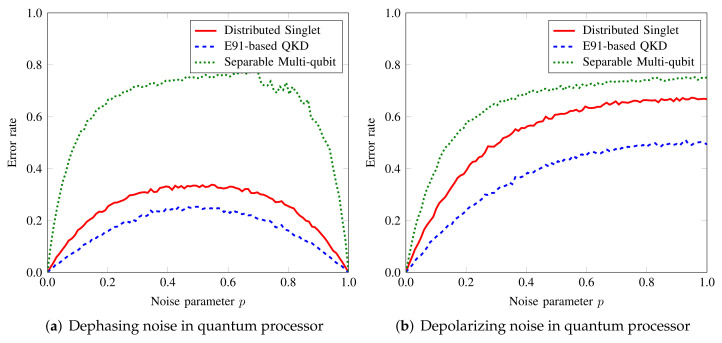
Impact of local node (Bob${}_{2}$)’s processor (**a**) dephasing and (**b**) depolarizing noise on the list distribution in tripartite network.

Figure 4a,b show the case of dephasing and depolarizing noise in the local processor’s (e.g., Bob${}_{2}$’s) memory, respectively. For dephasing noise, the effects are very similar to the case of desynchronization in phase definition. Again, the noise has the most adverse impact on the multiqubit case because each particle in the multiqubit state undergoes local noise. The distributed singlet performs best as the noise parameter increases because each party receives only a single particle from the server. At each processor, the noise becomes accumulated since the qubit is measured only at the last node. For QKD-based QBA, the nodes wait till they receive an acknowledgment from all parties. The additional duration for which qubit resides at the node increases the error rate for QKD compared to singlet; however, the trend for the two is the same. We have a similar tendency for depolarizing noise due to the same reason mentioned for dephasing noise. The error rate in list distribution is sensitive to a small value of depolarizing noise parameter, while it tapers off for larger noise values.

**Figure 5:**
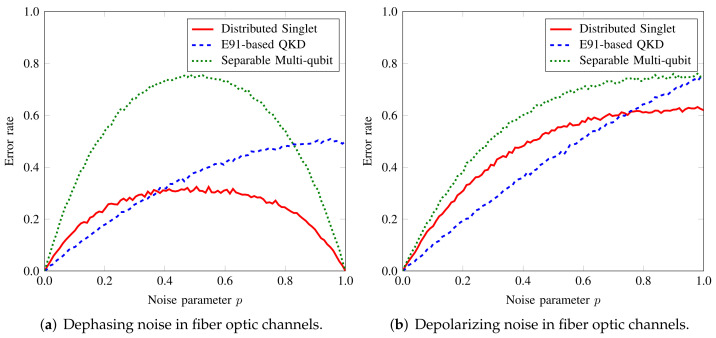
Impact of the optic fiber (**a**) dephasing (**b**) depolarizing noise on the list distribution in tripartite network.

Figure 5a,b show the case of dephasing and depolarizing noise on the qubits as they traverse over the fiber-optic channels. The noise performance is very similar to the scenario of local memory noises, as the models are essentially the same. Again, the noise has the most adverse effect on the multiqubit case because the qubits have to travel from one party to the next. For each noisy link, the noise gets accumulated since the qudit is measured only at the last node. For singlet case, the increase in noise makes the trend more linear. Since we consider all quantum channels to be noisy, more qubits will undergo quantum noise, as apparent in comparing figures for local and channel noise.

For the QKD case, Alice sends one qubit to each of the remaining K−1 parties, and then each party sends K−1 classical messages for broadcasting its basis choice. Therefore, the quantum communications per pass for QKD-based list distribution are K−1, while K(K−1) classical messages per pass. Similarly, for the multiqubit-based scheme (with $\left\lceil \log_{2}K\right\rceil$ qubits), it is $\left(K-1\right)\left\lceil \log_{2}K\right\rceil$ quantum and K(K−1) classical messages per pass, and for the singlet-based case with an external source, it is *K* and K(K−1) quantum and classical messages per pass, in accordance with [11].

## 6. Conclusions

In this paper, we have evaluated both entangled and entanglement-free QBA algorithms for practical blockchain-enhanced distributed networks, such as sensor networks and modular quantum computing. We considered these algorithms’ decentralization, security, and scalability and compared them for practical consensus. We observe that in general, quantum consensus algorithms have better fault-tolerance, lower scalability, and comparable decentralization compared to classical consensus algorithms. In the absence of quantum noise, entanglement-based consensus algorithms provide better security but do not scale very well. Though more scalable than other QBAs, the entanglement-free scheme offers less security and lesser decentralization because a single party performs measurement. In the presence of quantum decoherence, the local and/or fiber-optic quantum noise increases the generated list’s error rate. The effect of local noise is more pronounced on multiqubit QBA because it degrades all the qubits at the noisy node, unlike the entanglement-based schemes, where the noise degrades only those qubits that the node holds. Therefore, in quantum noise at a single node, entanglement-free QBA scheme is more affected than its counterparts. While in the case of noisy quantum channels, the effect is more comparable if all optical links have the same performance. We observed that the error rate in list distribution is susceptible to the small levels of local qubit noise and channel decoherence. Therefore, we infer that the current quantum protocols with NISQ devices and noisy quantum communication can only be employed in close-range small-scale distributed sensor networks and modular computing units.

## Figures and Tables

**Table 1:** System Setup Parameters.

Parameters	QKD	Singlet State	Qudit
Quantum State	two copies of entangled state	four-qubit singlet state	multiqubit state
Third-Party Source	**✗**	**✔**	**✗**
Classical Connections	pairwise authenticated channel	pairwise authenticated channel	pairwise authenticated channel
Quantum Connections	two quantum channels from Alice	pairwise from source to all parties	chain connection from first to last party
Quantum Processor Capability	Hadamard gate, CNOT gate, measurement in Pauli-Z or Pauli-X basis	measurement in Pauli-Z or Pauli-X basis	Hadamard, U, and V gate, measurement in Fourier basis

## Data Availability

Not applicable.

## Abbreviations

The following abbreviations are used in this manuscript:

SRF	Shared phase reference
BA	Byzantine Agreement
GHZ	Greenberger–Horne–Zeilinger
QBA	Quantum Byzantine Agreement
NISQ	Noisy intermediate-scale quantum
